# A269 SEX-DEPENDENT EFFECT OF PANCREASTATIN INHIBITION ON COLON MUCOSAL INTEGRITY IN HOMEOSTATIC CONDITION

**DOI:** 10.1093/jcag/gwad061.269

**Published:** 2024-02-14

**Authors:** D M Tshikudi, H Hitchinson, F Hesampour, J Ghia

**Affiliations:** University of Manitoba Faculty of Health Sciences, Winnipeg, MB, Canada; Immunology, University of Manitoba Max Rady College of Medicine, Winnipeg, MB, Canada; Immunology, University of Manitoba Max Rady College of Medicine, Winnipeg, MB, Canada; Immunology, University of Manitoba Max Rady College of Medicine, Winnipeg, MB, Canada

## Abstract

**Background:**

Sex bias in inflammatory bowel disease (IBD)-associated colorectal cancer (CRC) epidemiology is well known. Pancreastatin (PST), an epithelial cells-derived peptide overexpressed in IBD patients, regulates the endoplasmic reticulum (ER) and survival signalling involved in CRC. Pancreastatin inhibitor 8 (PSTi8), an inhibitor of PST, showed promising anti-inflammatory effects in IBD male mice model. However, no data exists regarding PST’s role in colon mucosal regeneration and epithelium maintenance.

**Aims:**

Here, we investigated the impact of PSTi8 treatment on colonic epithelial cells in male and female mice under homeostatic conditions.

**Methods:**

Male and female C57BL/6 mice were treated with PSTi8 (2.5mg/mL/kg, i.r.) or PBS for six days. Epithelial cells’ proliferation (Ki67) and differentiation (cytokeratin-20) were measured by immunofluorescence. Genetic markers for differentiated epithelial cells (goblet Muc2 and Tuft Dclk1), stem cells (fast-cycling Lgr5, reserve Hopx and fetal-like Ly6a), stem cell regulator (BMP4), epithelial cells lineage commitment (Atoh1, Hes1, Gfl1), ER activation (ATF6), and apoptosis (CHOP) were tested by qRT-PCR.

**Results:**

PSTi8 treatment decreased epithelial cell proliferation in both sexes. The inhibition of PST lowered the differentiation of epithelial cells and mucus production and increased tuft cell gene expression in females but not in male mice. In females, PSTi8 treatment increased fast-cycling stem cells but lower reserve and fetal-like stem cells in both sexes. In addition, PST inhibition significantly downregulated BMP4, which regulates epithelial cell differentiation, in females while not altering its expression in males. In contrast, PSTi8 treatment significantly increased transcription factors regulating epithelial cells' self-renewal and lineage commitments Hes1 and GFI1 in females but decreased Atoh1 in both sexes. PST inhibition enhanced gene expression of ER stress molecules ATF6 and CHOP in female and ATF6 in male mice. CHOP remained unaltered by PST inhibition in males.

**Conclusions:**

PSTi8 alters colonic epithelial regeneration, differentiation, and thus maintenance in a sex-dependent manner.

**Funding Agencies:**

CIHRChildren’s Hospital Research Institute of Manitoba (CHRIM)/Research Manitoba

